# A detailed protocol for expression, purification, and activity determination of recombinant SaCas9

**DOI:** 10.1016/j.xpro.2022.101276

**Published:** 2022-04-05

**Authors:** Franziska Flottmann, Greta Marie Pohl, Jan Gummert, Hendrik Milting, Andreas Brodehl

**Affiliations:** 1 Erich and Hanna Klessmann Institute, Heart and Diabetes Center NRW, University Hospital of the Ruhr-University Bochum, Georgstrasse 11, 32545 Bad Oeynhausen, Germany; 2 Department of Thoracic and Cardiovascular Surgery, Heart and Diabetes Center NRW, University Hospital Ruhr-University Bochum, Georgstrasse 11, 32545 Bad Oeynhausen, Germany

**Keywords:** CRISPR, Protein Biochemistry, Protein expression and purification

## Abstract

Recombinant SaCas9 is useful for a broad range of applications in the context of genome editing, especially when the specific protospacer adjacent motifs of other Cas9 derivatives are missing. Here, we describe a detailed protocol for the expression and purification of recombinant SaCas9. We detail the main steps for immobilized metal affinity chromatography and size exclusion chromatography. In addition, we present an assay for activity determination of SaCas9. Active SaCas9 can be purified in a week by using this protocol.

## Highlights

• Protocol for time and cost-effective expression and purification of recombinant SaCas9
• Details all steps necessary for performing chromatography
• Instructions for activity determination of purified recombinant SaCas9 by cleavage assay

## Graphical abstract

**Figure:**
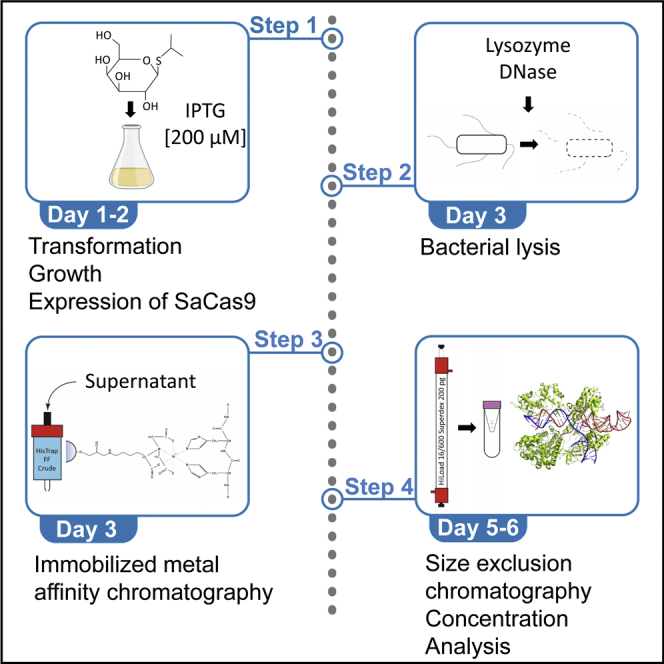

## Before you begin

Since the development of clustered regularly interspaced short palindromic repeat (CRISPR) Cas9 genome editing (Jinek et al., 2012), it received enormous interest in different life science areas. Usage of ribonucleoprotein complexes (RNPs) is of high interest, since no further genetic material with adverse effects must be introduced. However, the efficient expression and purification of recombinant Cas9 are limiting the use of RNPs in several applications. Some protocols for the expression and purification of Cas9 from *Staphylococcus pyogenes* (SpCas9) have been published (Rajagopalan et al., 2018; Carmignotto and Azzoni, 2019; Murugan et al., 2021). However, SaCas9 has a smaller molecular weight, a different molecular structure and uses a different protospacer adjacent motif (PAM, 5′-NNGRRT-3′) compared to SpCas9 (Nishimasu et al., 2015). Therefore, SaCas9 might be a useful alternative for genome editing if a PAM for SpCas9 is unavailable within the target DNA sequence.

### Transformation of One Shot BL21 star (DE3)

**Timing: 2 h**

1. Thaw chemically competent *Escherichia coli* (One Shot BL21 Star (DE3), Thermo Fisher Scientific, Waltham, MA, USA; C601003) for 10 min on ice.
2. Pipette 10 ng of the plasmid pET28-SaCas9 (Addgene, Teddington, UK; #178901) directly into the bacterial suspension. Mix the bacteria and the plasmid gently by flicking the tube. Do not vortex.
3. Incubate the mixture on ice (30 min). Afterwards, incubate the bacteria for 30 s at 42°C and put them directly on ice.
4. Add 250 μL of pre-warmed sterile LB-medium and incubate for 1 h at 37°C in a shaker.
5. Pre-warm agar plates containing 50 μg/mL Kanamycin sulfate.
6. Spread 50 μL of the bacterial suspension on an agar plate.
7. Incubate at 37°C for 12–16 h.

**Pause point:** After transformation, the agar plate can be stored for approximately four weeks at 4°C.

## Key resources table

**Table:** 

REAGENT or RESOURCE	SOURCE	IDENTIFIER
**Antibodies**		

Monoclonal mouse anti SaCas9 antibody (0.5 μg/mL)	GenScript	Cat#11C12
HRP conjugated goat anti mouse Ig antibody (1:1000)	BD Biosciences	Cat#554002

**Bacterial and virus strains**		

One Shot BL21 Star (DE3) chemically competent bacteria (*Escherichia coli*)	Thermo Fisher Scientific	Cat#C601003

**Chemicals, peptides, and recombinant proteins**		

Kanamycin sulfate	Sigma-Aldrich	Cat#K1377-1G
IPTG	Sigma-Aldrich	Cat#I6758-1G
LB-agar	Carl Roth	Cat#X965.2
LB-medium	Carl Roth	Cat#X968.1
Turbo DNase	Thermo Fisher Scientific	Cat#AM2238
Lysozyme	SERVA Electrophoresis	Cat#28263.02
Proteinase K	QIAGEN	Cat#19131

**Critical commercial assays**		

Pierce 660 nm Protein Assay Reagent	Thermo Fisher Scientific	Cat#22660
GeneJET PCR Purification Kit	Thermo Fisher Scientific	Cat#K0702
Trans-Blot Turbo Transfer Pack	Biorad	Cat#1704158
WesternBright Quantum HRP substrate	Advansta	Cat#K-12042-D20
MEGAshortscript T7 Transcription Kit	Thermo Fisher Scientific	Cat#AM1354

**Oligonucleotides**		

Primer *LMNA* Forward 5′-TTTCTCTCTTAGCAGAGTACCTAC-3′	This paper	N/A
Primer *LMNA* Reverse 5′-CCTACTCCATGAGCCACTAC-3′	This paper	N/A
Template In-Vitro-Transcription 5′-TCTCGCCAACAAGTTGACGAG ATAAACACGGCATTTTGCCTTGTTT TAGTAGATTCTGTTTCCAGAGTACT AAAACAAGTTGCTTCTTGGCCTCAC CCTATAGTGAGTCGTATTAAATT-3′	This paper	N/A
Primer In-Vitro-Transcription 5′-AATTTAATACGACTCACTATAGG-3′	This paper	N/A

**Recombinant DNA**		

pET28-SaCas9	This paper	Addgene Plasmid Cat#178901

## Materials and equipment

**Table:** Lysis buffer

Reagent	Final concentration
Tris-HCl	20 mM
NaCl	300 mM
Imidazole	25 mM
Tris(2-carboxyethyl)phosphine hydrochloride (TCEP)	0.1 mM
Phenylmethylsulfonyl fluoride (PMSF)	1 mM
DNase	20 μg/mL
Lysozyme	1 mg/mL

Check pH and adjust to 7.5. Store at 4°C for up to a week and pass through a sterile filter (0.2 μm) directly before use.

**Table:** Washing buffer (for immobilized metal affinity chromatography, IMAC)

Reagent	Final concentration
Tris-HCl	20 mM
NaCl	300 mM
Imidazole	25 mM
TCEP	0.1 mM

Check pH and adjust to 7.5. Store at 4°C for up to a week and pass through a sterile filter (0.2 μm) directly before use.

**Table:** Elution buffer (for IMAC)

Reagent	Final concentration
Tris-HCl	20 mM
NaCl	300 mM
Imidazole	300 mM
TCEP	0.1 mM

Check pH and adjust to 7.5. Store at 4°C for up to a week and pass through a sterile filter (0.2 μm) directly before use.

**Table:** Sample buffer (for size exclusion chromatography, SEC)

Reagent	Final concentration
Tris-HCl	20 mM
NaCl	300 mM
TCEP	0.1 mM

Check pH and adjust to 7.5. Store at 4°C for up to a week and pass through a sterile filter (0.2 μm) directly before use.

**Table:** Coomassie-R250 solution (for Coomassie staining)

Reagent	Final concentration
Coomassie-R250	0.25% w/v
Ethanol	30% v/v
Acetic Acid	10% v/v

This buffer can be stored at 20°C for several months.

**Table:** De-stain solution (for Coomassie staining)

Reagent	Final concentration
Ethanol	40% v/v
Acetic Acid	10% v/v

This buffer can be stored at 20°C for several months.

**Table:** Tris-buffered saline supplemented with Tween-20 (TBST, for Western blot analysis)

Reagent	Final concentration
Tris-HCl	20 mM
NaCl	100 mM
Tween-20	0.1% v/v

Check pH and adjust to 7.5 using HCl. Store at 20°C for up to two weeks.

**Table:** 2× Sample Buffer (for UREA-PAGE)

Reagent	Final concentration
Tris	90 mM
Boric acid	90 mM
EDTA	2 mM
Urea	7 M
Ficoll 400	12% m/v
Bromophenol blue	0.04% m/v
Xylene cyanol	0.04% m/v

Store at −20°C for several months.

**Table:** 10× Reaction buffer (for activity assay)

Reagent	Final concentration
Tris-HCl	50 mM
NaCl	100 mM
MgCl_2_	10 mM
Recombinant albumin	100 μg/mL

Check pH, adjust to 7.9, and store at −20°C for several months.

## Step-by-step method details

### SaCas9 expression

**Timing: 2 days**

After transformation of pET28-SaCas9 into One Shot BL21 Star (DE3) bacteria, the expression of recombinant SaCas9 can be induced by adding isopropyl-β-D-thiogalactopyranoside (IPTG).

1. Inoculate a single colony of the transformed One Shot BL21 Star (DE3) bacteria in 5 mL LB-medium supplemented with 50 μg/mL Kanamycin sulfate and incubate in a shaker at 37°C for 12–16 h.
2. Transfer the pre-culture into 1 L LB-medium supplemented with 50 μg/mL Kanamycin sulfate and shake the canonical flask vigorously at 37°C.
3. Measure the optical density at 600 nm (OD_600_) every 30 min. When the OD_600_ reaches a value of 0.5–0.6 (4–6 h), add IPTG (final concentration 200 μM) and incubate for 12–16 h at 18°C with vigorous shaking.
4. Centrifuge the bacteria for 20 min at 2,500 × *g* and 4°C. Remove the supernatant.

**Pause point:** Store the bacteria pellet at −80°C for up to two weeks.

### Lysis of bacteria

**Timing: 2 h**

**Figure 1:**
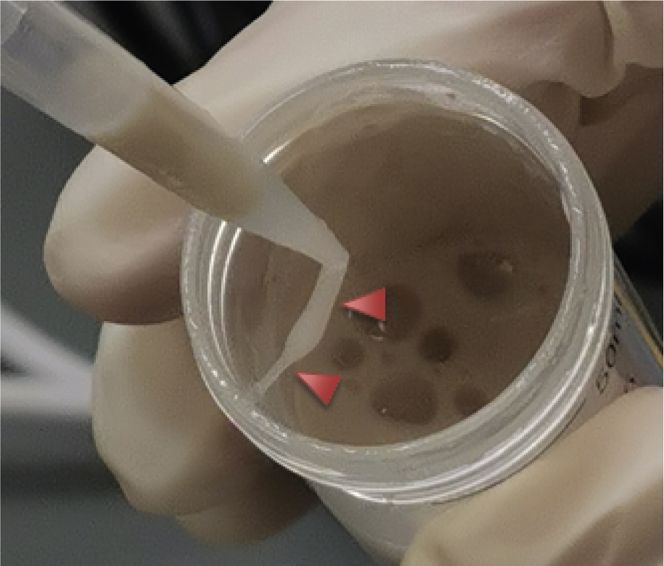
Slime formation using lysis buffer without DNase

The bacteria can be efficiently lysed by a combination of lysozyme and repeated freeze-thaw cycles. DNase should be added to prevent slime formation by the bacterial DNA (see Figure 1).

5. Thaw the frozen bacteria pellet on ice and re-suspend in 50 mL lysis buffer until no cell clumps are visible.
6. Incubate the lysis solution on ice for about 30 min.
7. Freeze the lysis solution using liquid nitrogen and thaw it at 30°C in an ultrasonic bath (8 min). Repeat this step five times in total.
8. Centrifuge the lysis solution for 20 min at 4°C and 2,500 × *g*.
9. Transfer the supernatant carefully to a fresh tube and repeat the centrifugation for another 20 min at 4°C and 2,500 × *g*.
10. Collect samples of the pellet and the supernatant (30 μL) for analysis by sodium dodecyl sulfate polyacrylamide gel electrophoresis (SDS-PAGE).
11. Filter the supernatant of the second centrifugation step using a syringe filter (0.45 μm) and continue directly with IMAC.

### Immobilized metal affinity chromatography

**Timing: 2 h**

The cDNA of SaCas9 is fused in the plasmid pET28-SaCas9 with a hexa-histidine tag (His_6_-Tag) at the C-terminus. Therefore, recombinant SaCas9 can be purified from the lysate by IMAC. Using a stepwise increase of imidazole will elute the bound SaCas9.

12. Perform an IMAC using the ÄKTApurifier system (Cytiva) or an equivalent system in combination with a 1 mL HisTrap FF Crude column (Cytiva). Use 1 mL/min flow-rate for all steps of the IMAC. Degas all buffers before use.
13. Wash the column with double distilled water (5 column volumes, CVs). Equilibrate the column with five CVs washing buffer containing 25 mM imidazole.
14. Use a superloop for sample loading (∼40 mL) onto the column (Figure 2A). Collect a sample of the flow-through (30 μL) for analysis by SDS-PAGE.

**Figure 2:**
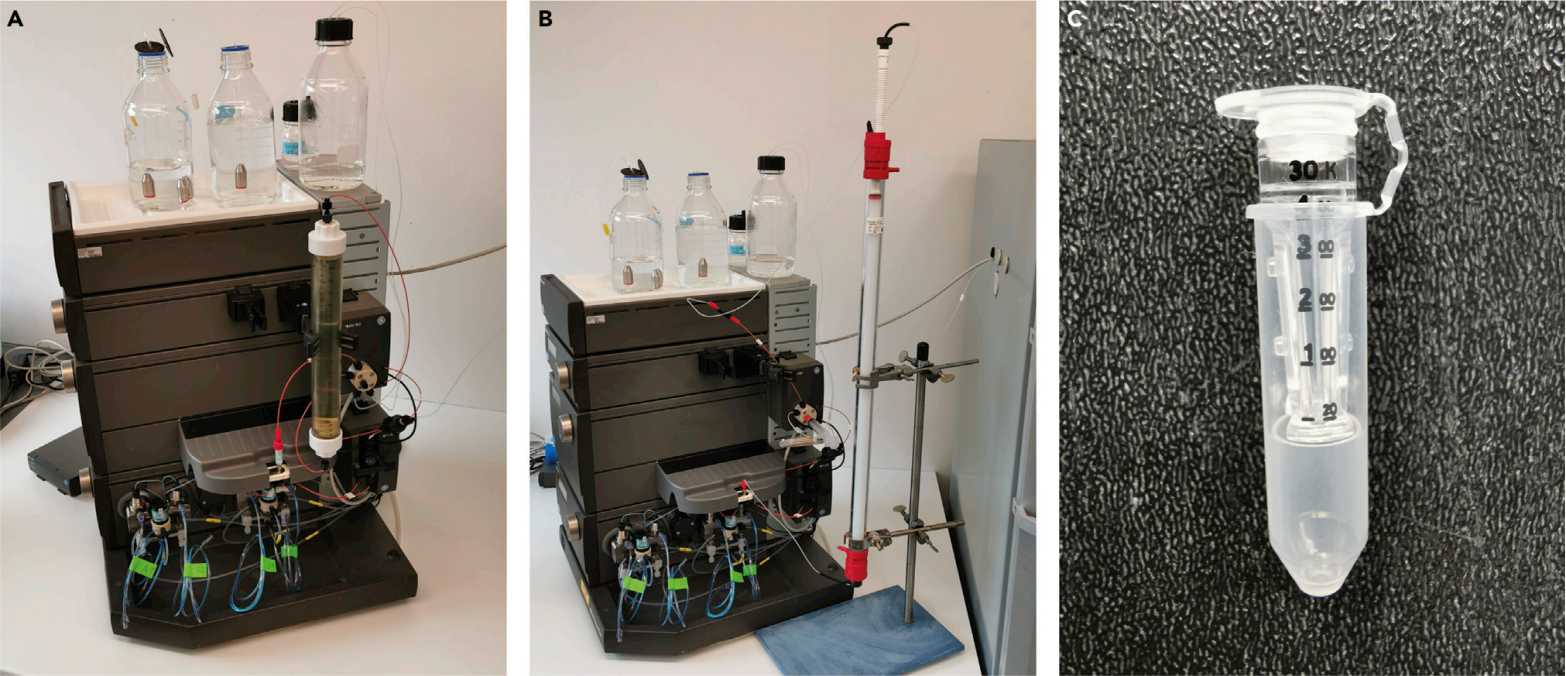
Chromatography system (A–C) Image of the Äkta system in combination with the superloop and the HisTrap FF Crude column (A) or the 5 mL loop in combination with the HiLoad 16/600 Superdex 200 Prep Grade column (B). Image of the centrifuge filter (C).
15. Wash the column with 20 CVs washing buffer and collect four fractions (5 mL).
16. Elute the SaCas9 with 12 CVs 60% elution buffer and 40% washing buffer (final concentration of imidazole 190 mM). Collect 1 mL fractions during elution. The percentage of the elution buffer was established based on a continuous gradient. However, to keep the volume as low as possible for direct application in SEC, we recommend a stepwise elution.
17. Clean the column with 10 CVs elution buffer (100%) and wash with five CVs double distilled water. Apply five CVs 20% v/v ethanol for storage of the column.
18. Analyze the fractions of the IMAC using SDS-PAGE (8%) in combination with Coomassie-R250 staining (Figure 3).

**Figure 3:**
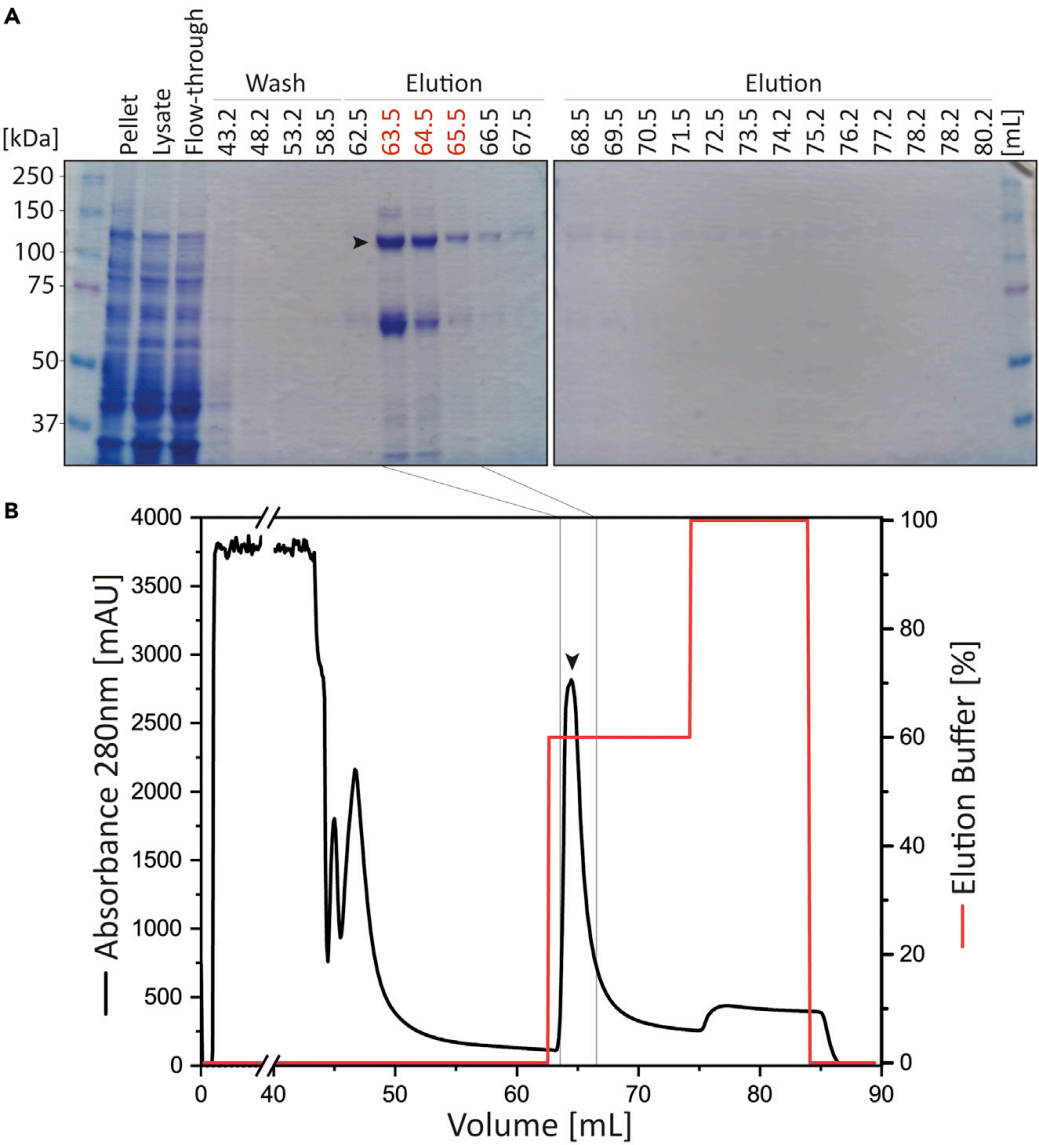
Purification of recombinant SaCas9-His_6_ by IMAC (A) SDS-PAGE and Coomassie-R250 staining of the fractions of the IMAC. The elution fractions (63.5–65.5 mL) (red) were pooled and used for SEC. Recombinant SaCas9 is marked with an arrow. (B) Chromatogram showing the elution of recombinant SaCas9 from the HisTrap FF Crude column. The further used fractions are indicated with an arrow.
19. Pool the fractions containing recombinant SaCas9 (∼128 kDa).

**Pause point:** Store the recombinant SaCas9 at 4°C for 12–16 h and continue on the next day with the SEC.

### Size exclusion chromatography

**Timing: 1 day**

SEC can be used to remove further protein contaminations. The described buffer system is used without any dialysis or buffer exchange between IMAC and SEC.

20. The ÄKTApurifier system in combination with a HiLoad 16/600 Superdex 200 Prep Grade column (Cytiva) can be used for SEC. Degas all buffers before use. Wash the column with four CVs double distilled water and equilibrate the column with two CVs sample buffer at flow-rate of 1 mL/min for 12–16 h.
21. Use a 5 mL sample loop for loading of the pooled IMAC fractions (∼3 mL, Figure 2B). Keep the volume as small as possible. Wash the loop with 25 mL sample buffer.
22. Purify recombinant SaCas9 with one CV sample buffer at 0.3 mL/min flow-rate for optimal resolution. Collect 1 mL fractions during the elution.
23. Analyze the fractions of the SEC using SDS-PAGE (8%) in combination with Coomassie-R250 staining and pool the fractions containing recombinant SaCas9 (Figure 4). Do not include fractions containing significant protein contaminations.

**Figure 4:**
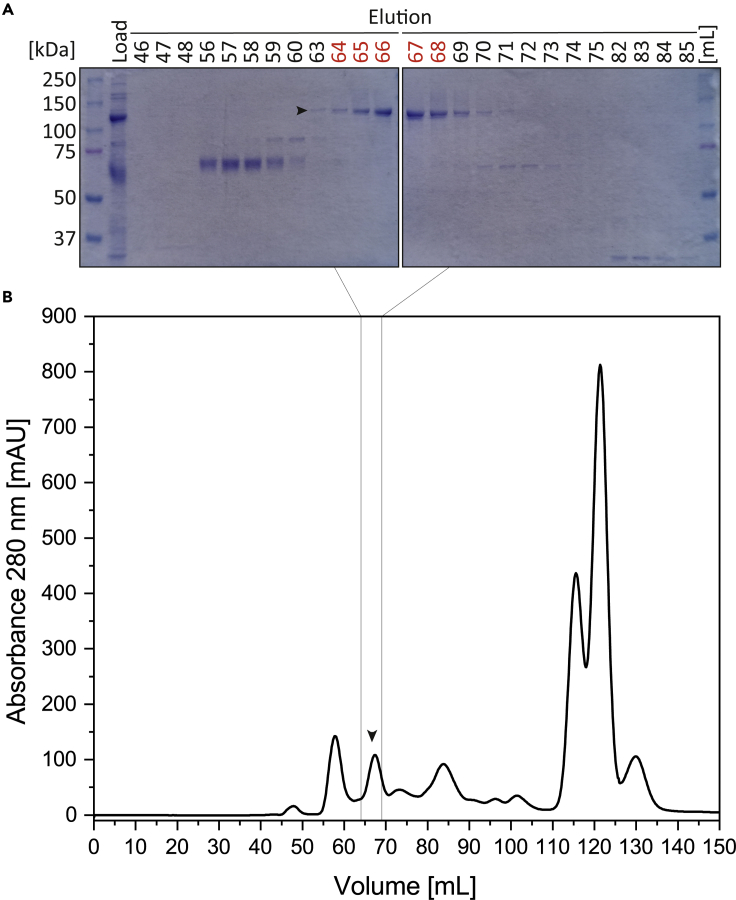
Purification of recombinant SaCas9-His_6_ by SEC (A) SDS-PAGE and Coomassie-R250 staining of the fractions of the SEC. Recombinant SaCas9 is marked with an arrow. (B) Chromatogram showing the elution of recombinant SaCas9 from the HiLoad 16/600 Superdex 200 pg column. The further used fractions (64–68 mL) are indicated with an arrow.
24. Clean the column with one CV sample buffer and wash it with four CVs double distilled water. Apply four CVs 20% v/v ethanol for storage of the column at 1 mL/min flow-rate.

### Protein concentration and buffer exchange

**Timing: 2 h**

The purified recombinant SaCas9 can be concentrated using centrifuge filters (Figure 2C) and stored at −20°C.

***Optional:*** Concentrate the eluted recombinant SaCas9 using Amicon Ultra 0.5 mL centrifuge filter units (30 kDa molecular cutoff).

25. Determine the protein concentration(s) using the Pierce 660 nm Protein Assay (Thermo Fisher Scientific) or an alternative protein assay according to the manufacturer’s instructions (https://www.thermofisher.com/document-connect/document-connect.html?url=https%3A%2F%2Fassets.thermofisher.com%2FTFS-Assets%2FLSG%2Fmanuals%2FMAN0011636_Pierce_660nm_Protein_Asy_UG.pdf) in combination with the M1000 Infinite plate reader (Tecan).
26. Add 50% v/v glycerin and mix gently.

**CRITICAL:** Since the high viscosity, calculate and weight the exact amount of glycerin instead of pipetting (ρ=1.26 g/mL). Addition of glycerin is highly recommended to prevent protein precipitation during freezing.

**Figure 5:**
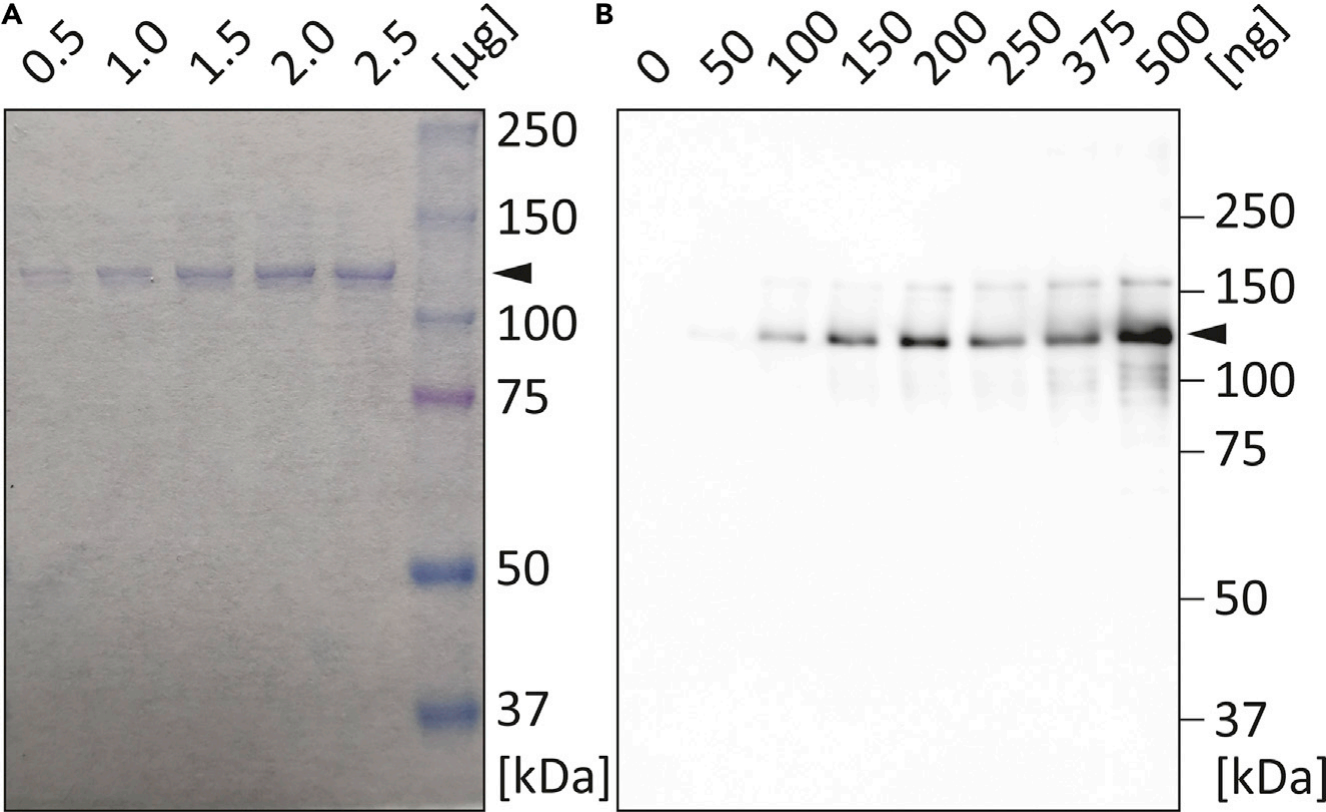
Analysis of recombinant SaCas9-His_6_ (A) SDS-PAGE and Coomassie-R250 staining of purified SaCas9 at specific quantities. Of note, the purity of the recombinant SaCas9 should be at least 90%. Recombinant SaCas9 is marked with an arrow. (B) Western blot analysis of recombinant SaCas9 (arrow) using primary monoclonal mouse anti SaCas9 antibodies.

27. Run a SDS-PAGE (8%) in combination with Coomassie-R250 staining with defined amounts of recombinant SaCas9 (50–2,500 ng) and determine the purity using e.g., ImageJ (Figure 5A).

**Pause point:** Store the purified recombinant SaCas9 at −20°C in appropriate aliquots until use.

### Western blot analysis

**Timing: 2 days**

The expression of the purified recombinant SaCas9 should be verified before performing any down-stream applications. The provided protocol can be used to detect SaCas9 by Western blot analysis. Instead, mass spectrometry can be used.

28. Run a SDS-PAGE (8%) of recombinant SaCas9 (50–500 ng per lane).
29. Transfer the proteins on a Trans-Blot Turbo Transfer Pack (Biorad) membrane using the Trans-Blot Turbo System (Biorad) according to the manufacturer’s instructions (https://www.bio-rad.com/sites/default/files/webroot/web/pdf/lsr/literature/10000071567.pdf).
30. Incubate the membrane in 5% w/v skimmed milk powder in TBST for 1 h at 20°C.
31. Incubate the membrane for 12–16 h at 4°C with monoclonal mouse anti SaCas9 antibodies (0.5 μg/mL, in TBST supplemented with 5% w/v skimmed milk powder).
32. Wash the membrane at least five times with TBST.
33. Incubate the membrane for 1 h at 20°C with polyclonal goat anti mouse immunoglobulin antibodies conjugated with horseradish peroxidase (HRP, 1:5,000 in TBST supplemented with 5% w/v skimmed milk powder).
34. Wash the membrane at least five times with TBST.
35. Incubate the membrane in WesternBright Quantum HRP substrate according to the manufacturer’s instructions (https://products.advansta.com/WesternBright-Quantum-user-manual) and detect the chemiluminescence signal with an appropriate detection system (Figure 5B).

### SaCas9 activity assay

**Timing: 1 day**

The functional activity of the purified SaCas9 should be verified by an in vitro cleavage assay before using it in down-stream applications. Here, we provide an activity assay targeting exon-3 of the human *LMNA* gene (NM_005572.3). However, also specific other cleavage assays can be used to demonstrate the activity of the purified recombinant SaCas9.

36. Perform a polymerase chain reaction (PCR) using the two oligonucleotides 5′-TTTCTCTCTTAGCAGAGTACCTAC-3’ (*LMNA*_for) and 5′-CCTACTCCATGAGCCACTAC-3’ (*LMNA*_rev) in combination with human genomic DNA as a template.

***Note:*** Human genomic DNA can be isolated for example with the High Pure PCR Template Preparation Kit (Roche) from HEK293 cells or any other human cell line. The PCR should be done using a DNA polymerase with a proofreading function like *e.g.,* Phusion or Pfu polymerase.

**Table:** PCR reaction master mix

Reagent	Amount [μL]
DNA template (genomic DNA)	2.5
Phusion DNA Polymerase (Thermo Fisher Scientific)	0.5
*LMNA*_for (10 μM)	2.5
*LMNA*_rev (10 μM)	2.5
Dimethyl sulfoxide (Thermo Fisher Scientific)	2.5
dNTPs (each 10 mM)	1
10× High Fidelity Buffer (Thermo Fisher Scientific)	10
ddH_2_O	28.5

**Table:** PCR cycling conditions

Steps	Temperature	Time	Cycles
Initial Denaturation	98°C	30 s	1
Denaturation	98°C	10 s	35
Annealing	60.3°C	30 s	
Extension	72°C	30 s	
Final extension	72°C	10 min	1
Hold	4°C	Forever

37. Purify the PCR product (509 bp) using the GeneJET PCR Purification Kit (Thermo Fisher Scientific) according to the manufacturer’s instructions https://www.thermofisher.com/document-connect/document-connect.html?url=https%3A%2F%2Fassets.thermofisher.com%2FTFS-Assets%2FLSG%2Fmanuals%2FMAN0012662_GeneJET_PCR_Purification_UG.pdf Measure the concentration of the purified PCR product using the NanoDrop 2000 photometer or an alternative photometer.

**Pause point:** Store the purified PCR product at −20°C.

38. Perform an in vitro transcription using the two oligonucleotides 5′-TCTCGCCAACAAGTTGACGAGATAAACACGGCATTTTGCCTTGTTTTAGTAGATTCTGTTTCCAGAGTACTAAAACAAGTTGCTTCTTGGCCTCACCCTATAGTGAGTCGTATTAAATT-3’ (template oligonucleotide) and 5′-AATTTAATACGACTCACTATAGG-3’ (T7 primer) in combination with the MEGAshortscript T7 Transcription Kit (Thermo Fisher Scientific) according to the manufacturer’s instructions (https://www.thermofisher.com/document-connect/document-connect.html?url=https%3A%2F%2Fassets.thermofisher.com%2FTFS-Assets%2FLSG%2Fmanuals%2Ffm_1354.pdf).
  a. Anneal 1 μL of the template oligonucleotide (100 μM) with 1 μL of the T7 primer (100 μM) and heat for 5 min to 95°C using a thermocycler. Cool down to 20°C (Δ5°C / min).
  b. Dilute the annealed oligonucleotides to a final concentration of 0.5 μM with RNase free water.
  c. Perform an in vitro transcription according to the following table and incubate at 37°C for 4 h.

**Table:** In vitro transcription master mix

Reagent	Amount [μL]
ATP	2
UTP	2
GTP	2
CTP	2
Diluted and annealed oligonucleotides (0.5 μM)	6
T7-10×-Buffer (Thermo Fisher Scientific)	2
RNase free water	2
T7 Enzyme Mix (Thermo Fisher Scientific)	2
  d. Add 1 μL Turbo DNase (Thermo Fisher Scientific) and incubate at 37°C for 15 min.
  e. Add 115 μL RNase free water and 15 μL ammonium acetate solution (Thermo Fisher Scientific). Gently mix the reaction solution.
  f. Add 300 μL ethanol and mix. Incubate at −20°C for 15 min and centrifuge at 4°C at 10,000 × *g* for 15 min.
  g. Remove the supernatant. Do not touch the pellet with the pipet tip. Resuspend the sgRNA in 50 μL RNase free water.
    **CRITICAL:** Pipette all steps in a biosafety cabinet and prevent RNase contamination. Use RNase free tubes and wear gloves during all steps. Measure the concentration of the synthesized single guide RNA (sgRNA) using the NanoDrop 2000 or an alternative photometer.
    **Pause point:** Store the purified sgRNA in aliquots at −80°C.
    **CRITICAL:** Prevent freeze thawing cycles of the sgRNA.

39. Run an urea polyacrylamide gel electrophoresis (UREA-PAGE) at 100 V for about 2 h (Summer et al., 2009) using the following 2× sample buffer dye (90 mM Tris, 90 mM boric acid, 2 mM EDTA, 7 M urea, 12% m/v Ficoll 400, 0.04% bromophenol blue, 0.04% xylene cyanol) for quality control.
40. Incubate the gel for about 20 min with GelStar Nucleic Acid Gel Stain (1:5000, Lonza) at 20°C and detect the bands using a gel documentation system (Figure 6A).

**Figure 6:**
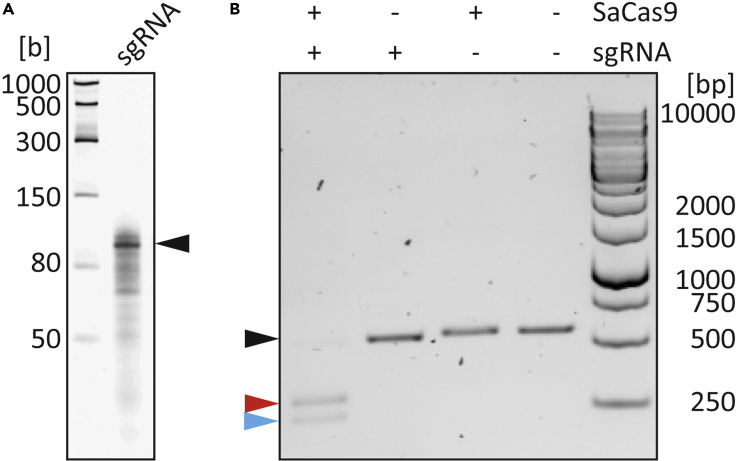
Activity of recombinant SaCas9-His_6_ (A) Urea-PAGE and staining of in vitro transcribed sgRNA (118 bases, black arrow). (B) Expected results of the SaCas9 activity assay. The black arrow indicates the uncleaved PCR product (*LMNA*, exon-3, NM_005572.3, 509 bp). The size of the expected cleavage products is 281 bp (red arrow) and 228 bp (blue arrow).
41. Mix 2.5 pmol (322 ng) of purified recombinant SaCas9 and 2.5 pmol of the sgRNA (95 ng) on ice.
42. Add 10-fold reaction buffer and complement with RNase-free water (total volume 10 μL). Incubate the samples for 10 min at 25°C. Add 0.2 pmol (62.9 ng) of the purified target PCR product and incubate for 1.5 h at 37°C followed by a heat inactivation at 80°C for 5 min.

**CRITICAL:** Pipet this step in a biosafety cabinet to prevent any contaminations with RNases and wear clean gloves. Perform three control reactions missing either recombinant SaCas9 or the sgRNA or both.

43. Add 1.5 μL proteinase K (600 mAU/mL) to all samples and incubate at 37°C for 20 min.
44. Perform an agarose gel electrophoresis (130 V, 40 min) to analyze the activity of SaCas9. Two DNA fragments with 281 and 228 bp are expected (Figure 6B).

## Expected outcomes

The first purification step using IMAC enriches recombinant His_6_-tagged SaCas9 (Figure 3). Since the volume should be kept as low as possible for the subsequent SEC, a stepwise elution with imidazole containing elution buffer is recommended (Figure 3B). However, several endogenous proteins from *E. coli* are also binding to the Ni^2+^-nitrilotriacetic acid (NTA) column (Figure 3A) indicating that a second purification step is indispensable.

SEC can be used to purify recombinant SaCas9 to homogeneity (Figure 4A). Several protein contaminations can be removed by SEC (Figure 4B). In addition, the imidazole from the IMAC is removed by this step. The eluted SaCas9 can be either directly used or concentrated using Amicon ultra centrifugation filters. It can be also stored with 50% v/v glycerin at −20°C.

A SDS-PAGE in combination with Coomassie-R250 staining can be used to determine the purity of recombinant SaCas9 at specific protein concentrations (Figure 5A). Western blot analysis using specific anti SaCas9 antibodies can be performed for identification of SaCas9 (Figure 5B). The activity of purified recombinant SaCas9 should be verified by targeting e.g., exon-3 of the *LMNA* gene (NM_005572.3, chr1:156084461–156107657, human hg19, Figure 6B).

## Limitations

With this protocol, it is possible to express and purify recombinant SaCas9 with a purity about 95%. This protocol has the advantage that IMAC and SEC can be directly performed without need of dialysis or buffer exchange. However, for crystallization studies further purification steps might be necessary. The activity of the purified recombinant SaCas9 can be proven by the described in vitro cleavage assay. However, success of genome-editing experiments also depend highly on the quality of the sgRNA and is dependent on the target sequence. Some genomic regions might be difficult to address by SaCas9 because of steric inaccessibility because of the chromatin structure.

## Troubleshooting

### Problem 1

During lysis of the bacteria, the bacterial DNA can increase the viscosity of the supernatants (see Figure 1) interfering with centrifugation steps and purification. This problem may rise in Step-by-step method details step 6.

### Potential solution

We highly recommended adding DNase to prevent slime formation by the genomic DNA.

### Problem 2

We observed several protein contaminations after the IMAC (see Figure 3), which might interfere with different down-stream applications. This problem may rise in Step-by-step method details step 18.

### Potential solution

We recommended performing a SEC to receive higher purity of recombinant SaCas9.

### Problem 3

Originally, a gradient elution with increasing imidazole concentrations was used in the IMAC. However, a gradient elution increases the volume of eluted recombinant SaCas9 interfering with the direct application of a SEC. This problem may rise in Step-by-step method details step 16.

### Potential solution

To prevent an additional concentration step, we recommend a stepwise elution with imidazole containing elution buffer as indicated (Figure 3B).

### Problem 4

**Figure 7:**
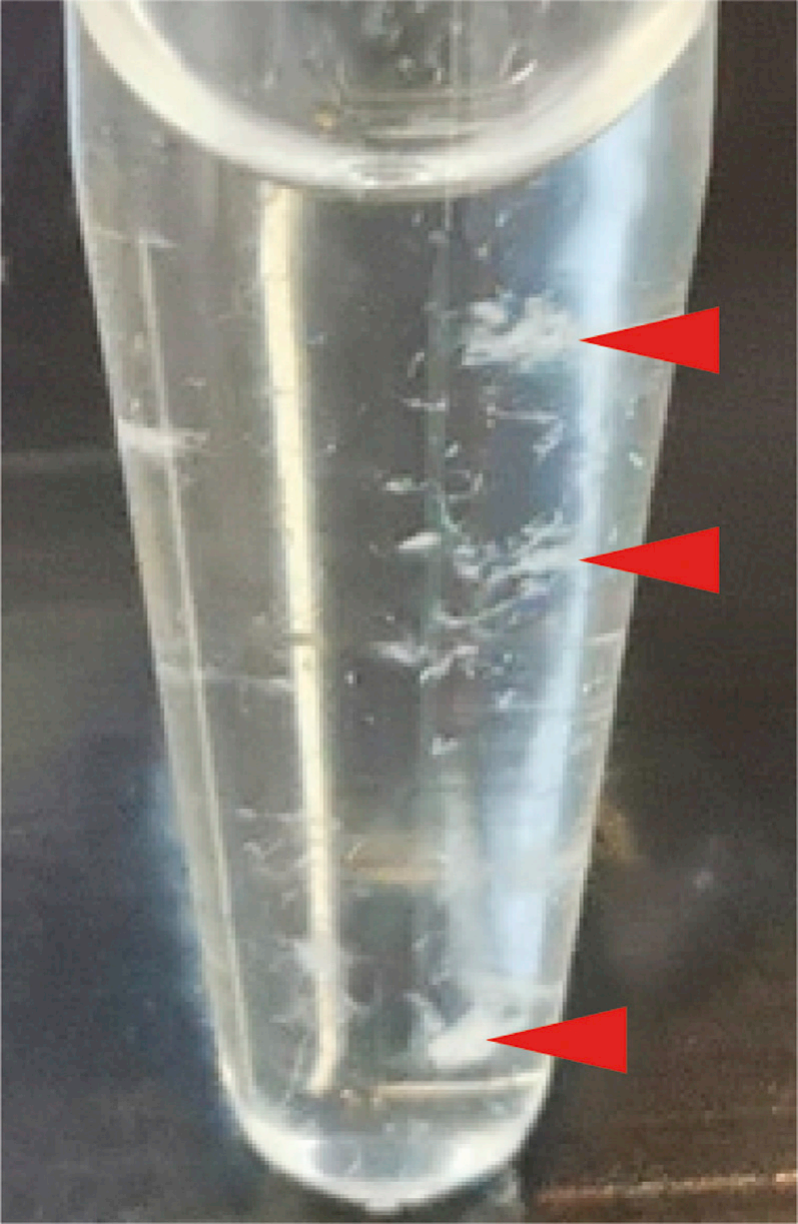
SaCas9 precipitates in buffers with low NaCl concentration

In buffers with low NaCl concentrations, we observed precipitation of recombinant SaCas9 (see Figure 7). This problem may rise in Step-by-step method details step 19.

### Potential solution

If you observe SaCas9 precipitation, check the NaCl concentration and use 300 mM in all buffers. In addition, the recombinant SaCas9 should be stored at −20°C supplemented with 50% glycerin to prevent protein precipitation by freezing and should be gently handled.

### Problem 5

The sgRNA might degrade if RNase contamination occurs. This problem may rise in Step-by-step method details step 39.

### Potential solution

If you observe significant degradation of the in vitro transcribed sgRNA, please repeat all steps in a biosafety cabinet and wear gloves during the complete procedure. Use RNase free tubes and pipet tips. Store all reagents in appropriate aliquots to prevent contamination of the stock solutions.

## Resource availability

### Lead contact

Further information and requests for resources and reagents should be directed to and will be fulfilled by the lead contact, Andreas Brodehl abrodehl@hdz-nrw.de.

### Materials availability

The plasmid pET28-SaCas9 (Supplemental information, Figure S1) has been deposited to Addgene (Teddington, UK; www.addgene.org, Cat#178901) or can be directly received from the corresponding author.

## Data Availability

The published article includes all data generated or analyzed during this study.

## Supplemental information

Figure S1. Plasmid map of pET28-SaCas9
