# Supplementary figures and images for: A detailed protocol for expression, purification, and activity determination of recombinant SaCas9
Source: STAR Protoc. 2022 Apr 5;3(2):101276. doi: 10.1016/j.xpro.2022.101276 (PMC8991255; doi:10.1016/j.xpro.2022.101276)

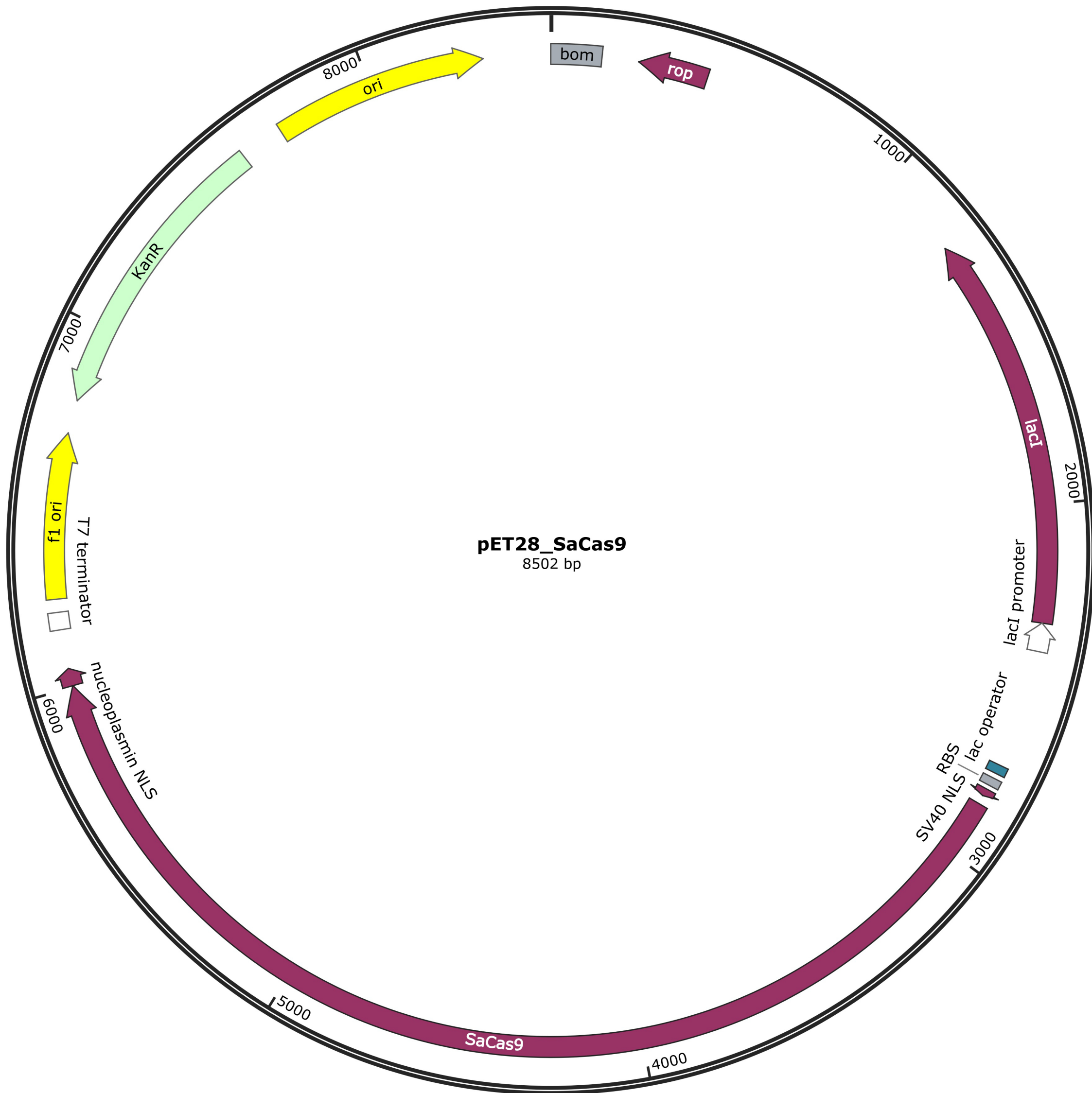

Supplement: Figure S1. Plasmid map of pET28-SaCas9 [file mmc1.pdf]
